# Kinetics of antibody-induced modulation of respiratory syncytial virus antigens in a human epithelial cell line

**DOI:** 10.1186/1743-422X-4-68

**Published:** 2007-07-03

**Authors:** Rosa E Sarmiento, Rocio G Tirado, Laura E Valverde, Beatriz Gómez-Garcia

**Affiliations:** 1 Departamento de Microbiología y Parasitología, Facultad de Medicina, Universidad Nacional Autónoma de México, Ciudad Universitaria, D.F., México

## Abstract

**Background:**

The binding of viral-specific antibodies to cell-surface antigens usually results in down modulation of the antigen through redistribution of antigens into patches that subsequently may be internalized by endocytosis or may form caps that can be expelled to the extracellular space. Here, by use of confocal-laser-scanning microscopy we investigated the kinetics of the modulation of respiratory syncytial virus (RSV) antigen by RSV-specific IgG. RSV-infected human epithelial cells (HEp-2) were incubated with anti-RSV polyclonal IgG and, at various incubation times, the RSV-cell-surface-antigen-antibody complexes (RSV Ag-Abs) and intracellular viral proteins were detected by indirect immunoflourescence.

**Results:**

Interaction of anti-RSV polyclonal IgG with RSV HEp-2 infected cells induced relocalization and aggregation of viral glycoproteins in the plasma membrane formed patches that subsequently produced caps or were internalized through clathrin-mediated endocytosis participation. Moreover, the concentration of cell surface RSV Ag-Abs and intracellular viral proteins showed a time dependent cyclic variation and that anti-RSV IgG protected HEp-2 cells from viral-induced death.

**Conclusion:**

The results from this study indicate that interaction between RSV cell surface proteins and specific viral antibodies alter the expression of viral antigens expressed on the cells surface and intracellular viral proteins; furthermore, interfere with viral induced destruction of the cell.

## Background

Antibody-induced modulation of antigen is a complex biological phenomenon closely resembling other receptor-ligand interactions. Following exposure to specific antibodies, surface antigens are usually redistributed on the cell surface and are internalized or expelled into the extracellular medium [1,2]. These phenomena have been widely reported in virus systems [3-5], the best studied being an alpha herpes; in pseudorabies [6-9]. In that system, following exposure to specific antibodies, cell-surface antigens are usually redistributed with the membrane-bound viral glycoproteins aggregating to form patches on the cell surface. In fibroblasts and epidermoid cells, the patches subsequently polarize to one area of the cell, thus producing caps that are shed into the extracellular space [6-9]. In contrast, in monocytes, glycoprotein patches do not form caps, but instead collect in regions of the plasma membrane which are specialized for internalization through clathrin-coated pits. After the clathrin coated pits are introduced into the cell, the antibody-antigen complexes are degraded and the glycoproteins are directed back to the plasma membrane [8-10].

Respiratory syncytial virus (RSV) is an enveloped pneumovirus classified within the *Paramyxoviridae *family. Its genome encodes two non-structural and nine structural proteins, three of which are transmembrane surface glycoproteins: The G protein is involved in the virus attachment; the F protein mediates fusion of virus with cell membranes [11], and SH protein inhibits TNF-alpha signalling [12]. Cells infected with RSV can fuse with adjacent cells resulting in giant multinucleated syncytium, polykaron formation besides being cytophatic favors virus spread [11].

Worldwide, RSV is the most important viral pathogen of serious lower-respiratory tract illness in infants and young children. RSV infects nearly 70% of infants in their first year of life; by the age of 24 months old virtually all children will have been infected at least once and about half will have experienced at least two infections [11,13,14]. RSV also causes significant disease in adults (especially those in contact with children); it is also regarded as an important cause of serious illness/morbidity occurring in the elderly [15] and in patients with a compromised immune system [16]. Severe RSV disease appears to be linked to an unbalanced immune response [14,17-19], it has also been associated with asthma [20-23] and acute exacerbations of chronic obstructive pulmonary disease (COPD) [24-26]. The mechanisms, by which this infection leads to airway dysfunction that persists long after the acute disease has been resolved, are not well defined. However, involvement of RSV persistence in long term respiratory problems has been suggested [18-20,24-29].

Individuals previously infected with RSV can be subsequently re-infected (within months) with either an identical or antigenically closely related virus despite the presence of serum antibodies [11,13,29]. RSV persistence has been postulated as a reservoir for viral transmission and re-infection [18,26]. Both the innate and adaptive immune responses participate in clearing the virus and the pathogenesis associated with infection [11,14,17-20,25,26]. In temperate climates, annual RSV outbreaks occur predictably from late fall to early spring [30,31],

The current study was designed to examine whether RSV-cell-surface-antigen-antibody complexes (RSV Ag-Abs) in epithelial cells undergo aggregation into patches that subsequently either form caps or are internalized through endocytosis. Furthermore, kinetic assays were used to determine the concentration level and fate of viral proteins in RSV-infected cells that had been incubated with anti-RSV antibodies.

By determining the RSV Ag-Abs in plasma membrane and viral proteins in the cytoplasm, we investigated the effect of the presence of RSV-specific IgG on infected epithelial cells over a period of time (1 hour) and on the viability of these cells. Here, we present evidence that anti-RSV IgG induced redistribution of cell surface viral glycoproteins and that internalization of RSV Ag-Abs was partially inhibited by incubation in hypertonic medium, thus suggesting the participation of a clathrin-mediated mechanism. We also observed a time-dependent, cyclic fluctuation in the concentration of RSV Ag-Abs in cell surface and in intra-cellular viral proteins. Moreover, anti-RSV IgG protected HEp-2 cells from viral-induced cell death.

## Methods

### All reagents were from Sigma, unless otherwise specified

#### Virus and cells

Human epidermoid carcinoma larynx cell line HEp-2 from our laboratory (originally from ATCC) was grown in Dulbecco's modified medium (D-MEM; GIBCO BRL 12100-038) which was determined to be mycoplasma free by using a mycoplasma detection kit (Boheringer Mannheim). Long strain RSV has been used as the prototype virus in our laboratory for over ten years. The procedures for propagating cells and viruses and for assaying viral infectivity have been described elsewhere [32].

#### Anti-RSV antibodies

Polyclonal anti-RSV sera were obtained in our laboratory from male New Zealand rabbits after three intramuscular immunizations with RSV (1 × 10^6 ^TCID_50_/ml; 400 μg protein/ml) that had been purified by linear sucrose gradient. Pre-immune sera was obtained from the rabbits before immunization. Anti-RSV serum characteristics were evaluated by its neutralization activity in viral infectivity [33], and by the presence of antibodies against RSV proteins. The specific antibodies against viral proteins were determined by western blot assays with the purified RSV virion utilized to immunize the rabbits. Proteins with apparent molecular weight from 45 to 240 kDa were detected. The presence of RSV glycoproteins was confirmed by flow cytometry assays to determine cell-surface RSV proteins in infected HEp-2 cells [34].

RSV IgG and pre-immune IgG were obtained according to Harlow and Lane, with some modifications [35]. Briefly, adding the serum through a protein-A sepharose column after keeping the column overnight a 4°C. IgG were eluted with acetic acid 0.1 M and NaCl 0.15 M and the fractions of 1 ml were collected in tubes with 100 μl of Tris-HCl buffer 8.0. Fractions with OD Of 0.9 to 2.07 at 280 nm were collected and the protein content was determined by Lowry. Polyclonal serum with viral infectivity neutralization titer of, 1.2 × 10^4 ^TCID_50_/ml per 30 μg protein/ml was used.

#### Visualization of RSV antigen

HEp-2 cells (1 × 10^4^) that had been grown on glass cover slips, previously treated with poly-L lysine (1 μg/ml at room temperature (RT)) and washed with phoshate-buffered saline (PBS), for 30 min., were infected with RSV at a multiplicity of infection (m.o.i.) of 50 (12 h; 37°C; 5% CO_2_-air) and thereafter washed in PBS. Infected cells were permeabilized and fixed with cold methanol (5 min) and cold acetone (30 sec) and the viral antigen was visualized by indirect immunofluorescence by using goat anti-RSV (MAB 858-1 Chemicon, Temecule, CA) as first antibody and rabbit anti-goat fluorescence conjugate (61–1611 Zymed, South San Francisco, CA) as second antibody, as previously described [32]. Fluorescence-labelled proteins were examined by confocal microscopy. As control, mock-infected cells were used.

#### Visualization of RSV Ab-Ags on the cell surface

To RSV-infected cells (three cover slips), anti-RSV IgG, diluted 1:5 in D-MEM containing 1% glutamine, was added to cover the cell monolayer and the mixture was incubated at 37°C. At 0, 10, 20, 30, 40, 50, or 60 minutes of incubation, cell samples were taken, then washed in PBS to remove excess of anti-RSV IgG, and fixed with 4% paraformaldehyde in PBS. The RSV Ag-Abs was visualized by indirect immunofluorescence as previously described. Controls were, RSV-infected cells (three cover slips) incubated with pre-immune polyclonal IgG, mock-infected cells (three cover slips) incubated with anti-RSV IgG and (three cover slips) with pre-immune polyclonal IgG.

#### Detection of intracellular RSV proteins

Cells grown in cover slips were infected and incubated at 37°C with anti-RSV IgG, at 0, 10, 20, 30, 40, 50, or 60 minutes washed permeabilized and fixed. Then intracellular viral proteins were detected by adding anti-RSV IgG and incubated 1 h at 37°C. Afterwards, cells were washed and viral proteins visualized by indirect immunofluorescence as described. Controls were as described for visualization of RSV Ab-Ags on the cell surface.

#### Inhibition of internalization of RSV Ag-Abs by hypertonic incubation

The sucrose inhibition assay was used [36]. Briefly infected cells, which had been grown on cover slips as described, were incubated (30 min; 37°C) with D-MEM supplemented with 2% fetal bovine serum (FBS) and 0.3 M sucrose. Then, anti-RSV IgG was added and were incubated in D-MEM containing 2% FBS and 0.3 M sucrose. At various incubation times (0, 10, 20, 30, 40, 50, or 60 min), cells were permeabilized and fixed, then after anti-RSV IgG was added and intracellular RSV proteins were determined as described above.

#### Confocal laser scanning microscopy

Fluorescent samples were examined in a Bio-Rad MRC 600 confocal laser scanning system that was linked to an Axioskop Zeiss microscope with Plan-Neuflour 40×/0.75P H2 objective. Krypton-argon laser light was used to excite fluorescein isothiocyanate (FITC; 488 nm line) with emission BHS filter. Data were processed with Bio-Rad CoMOS with Z-step of 1.08 nm.

#### Cell viability

To infected HEp-2 cells, anti-RSV IgG was added, and cell viability was determined at 2, 24, 48, and 72 h of incubation. Sterile 0.2% ethylenediamine tetra acetate (EDTA) in 0.9% saline was added to each cell monolayer; after incubation (15 min), the cells were suspended in D-MEM containing 2% FBS and trypsin (5 μg/ml). Trypan blue solution was added to the cell suspension and the viable cells were counted by light microscopy. As controls, both infected cells incubated with pre-immune IgG and mock-infected cells incubated with anti-RSV IgG were used.

## Results

### RSV proteins in infected HEp-2 cells

**Figure 1:**
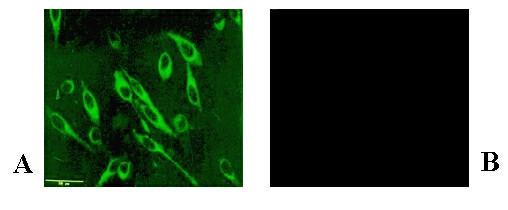
**Indirect immunofluorescence of RSV antigen in infected epithelial cells**. Viral proteins in HEp-2 cells, which had been infected at m.o.i. of 50 for 12 h permeabilized and fixed with acetone and cold methanol, were visualized by indirect immunofluorescence with an epifluorescent microscope. First antibody: goat anti-RSV; second antibody: rabbit anti-goat. A) RSV-infected cells; B) mock-infected cells.

RSV proteins, present in viral infected HEp-2 cells, were visualized in permeabilized cells by indirect immunofluorescence, at various times after infection. We observed (results not shown) that viral proteins could be visualized at 6 to 8 h post infection (p.i., fluorescence intensity of 1–2); however, for 90 to 95% of the cells, a fluorescence intensity of 2–3 was observed at 12 h p.i. (Fig. 1), yet neither cell destruction nor infective extracellular virus was found. At longer incubation times, fluorescence intensity increased, and cell destruction was evident; therefore, subsequent experiments were done with cells infected for 12 h p.i.

### Anti-RSV IgG induced redistribution of viral glycoproteins on the surface of infected cells

The interaction of RSV-specific IgG with cell surface RSV glycoproteins was determined by examining the binding of anti-RSV IgG to infected cells at 0, 10, 20, 30, 40, 50, or 60 minutes of incubation.

**Figure 2:**
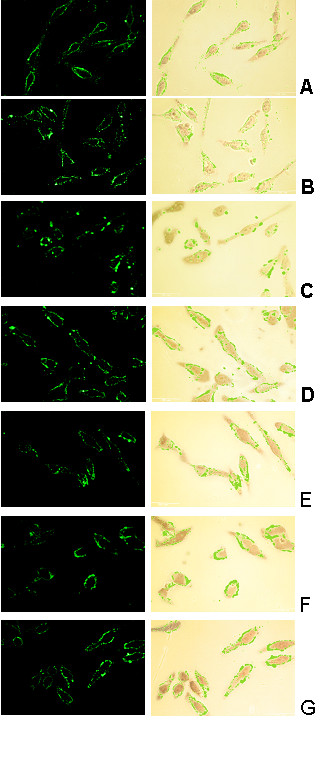
**Confocal laser scanning image of the distribution of viral glycoproteins on cell surface of epithelial cells**. HEp-2 cells had been infected at m.o.i. of 50 for 12 h then incubated with anti-RSV IgG and fixed, at different times, with paraformaldehyde. Kinetics of viral proteins determined according to material and methods. Incubation time (min):A) 0, control; B) 10;C) 20; D) 30; E) 40; F) 50; and G) 60. Images of fluorescein-labelled proteins: left with UV light, right with visible and UV light.

Antibody glycoproteins complexes on the plasma membrane of infected cells were visualized in parformaldehyde-fixed cells by indirect immunofluorescence assay, the fluorescently labelled cells were examined by confocal microscopy. Because the cells were not permeabilized, the labelled antibody detected only anti-RSV IgG antibody bound to glycoproteins on the cell surface (Fig. 2). Initial experiments were done at m.o.i. of 10 to 20; however, because the anti-RSV IgG induced rearrangement of RSV Ag-Abs was best observed at higher m.o.i. (50) therefore assays were done at that multiplicity.

The presence of fluorescent RSV glycoproteins on the cell surface was scored in the following manner: rim, when the florescence was homogenously distributed; patch, when the proteins were in randomly distributed aggregates; and caps, when the patches were polarized in a site on the cell. The intensity of fluorescence observed in the images from the immunofluorescence assays was arbitrary expressed, with 1 as the lowest intensity and 3, the highest. Results were obtained by examining the distribution of fluorescence on at least 100 cells. All assays were run independently at least three times.

In the 0-minute-incubation (anti-RSV IgG control) assay, antibody-bound viral glycoproteins were observed as a rim, with fluorescence intensity of 1 (Fig. 2A). After 10-minute incubation with antibody, RSV Ag-Abs complexes were aggregated as randomly distributed patches with fluorescence intensity of 2 (Fig. 2B). At 20-minute incubation, the fluorescence intensity increased to 3 and the antibody-glycoprotein complexes were found to be rearranged as caps on the cell surface; however, areas without detectable RSV proteins were present (Fig. 2C).

In the 30-minute-incubation assay, RSV Ag-Abs content and fluorescence intensity decreased, with the fluorescence (intensity of 2) being once again observed as rim (Fig. 2D). In the 40-minute-infection assay, the fluorescence was found basically on the rim, with intensity of 1 (Fig 2E). On further incubation (50 min), cells showed an intensity of 2, with antibody-bound glycoproteins as rim, with a few patches (Fig. 2F). In the 60-minute-incubation assay, the fluorescence had increased to an intensity of 3 and showed the viral proteins with antibody arranged as rim (Fig. 2G). Thus, over the course of the incubation, redistribution of viral glycoproteins varied and a cyclic fluctuation of RSV Ag-Abs concentration was observed.

### Intracellular RSV proteins concentration fluctuates with cyclic pattern with respect to incubation time

After establishing that anti-RSV IgG had induced an incubation-time-dependent fluctuation in the concentration of cell-surface RSV Ag-Abs, we decided to determine whether a similar effect would occur in intracellular viral proteins, therefore, anti-RSV IgG was added to permeabilized cells and observed by indirect immunoflourescence. Results were obtained by examining the distribution of fluorescence on at least 100 cells. All assays were run independently at least three times.

**Figure 3:**
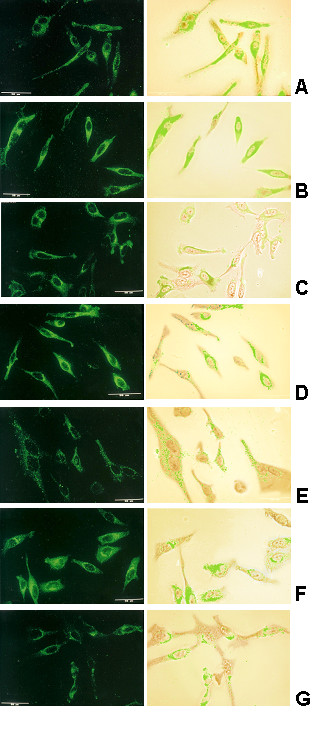
**Confocal laser scanning image of intracellular RSV proteins**. HEp-2 cells had been infected and then incubated for various times with anti-RSV IgG were permeabilized, fixed with acetone and methanol and anti-RSV added. The kinetics of intracellular viral proteins was determined as described in material and methods.

At time 0, the cytoplasm of permeabilized infected cells (Fig. 3A) was fluorescent (intensity of 1), implying that viral proteins were present. An increase in concentration of intra-cellular labelled proteins was evident after 10 minutes of incubation with antibody (intensity of 3) (Fig. 3B), suggesting that RSV Ag-Abs were internalized. On further incubation (20 minutes), the fluorescence intensity was found to be diminished (intensity of 1, Fig. 3C). At 30 minutes (Fig. 3D), the labelled proteins (flourescence intensity 2) were localized in cytoplasm areas, although cells without labelled proteins were observed.

At 40 minutes, a clear reduction in the concentration of viral proteins was evident, as the fluorescence was negligible (Fig. 3E), whereas a noteworthy increase (intensity 3, Fig. 3F) in the content of labelled RSV intracellular viral proteins was observed at 50-minute incubation. Finally, at 60 minutes, the protein concentration decreased and the labelled RSV proteins were localized in some areas of the cytoplasm (Fig. 3G). Moreover, infected cells without detectable labelled proteins were observed in samples from 20- to 60-minute incubation. These data show a time-dependent, cyclic fluctuation in the content of intracellular RSV proteins.

### Clathrin-dependent endocytosis contributed to internalization of RSV Ag-Abs

Our results showed that anti-RSV IgG incubation induced the removal and re-appearance of intracellular viral proteins in a cyclic manner (Fig. 3), an effect that might be related to caps expelled into the extracellular space or to internalization of antigen-antibody complexes. Internalization of receptor-ligand complexes (endocytosis) is mediated mainly by clathrin-coated pits, regions of the cell-surface membrane, which are specialized in the internalization process. Receptor-ligand complexes on the cell membrane accumulate in these regions [37]. Therefore, we decided to determine whether internalization of RSV Ag-Abs may be associated with clathrin-dependent endocytosis.

The strategy consisted of inhibition of receptor-mediated endocytosis by exposing the cells to hypertonic medium containing sucrose. Clathrin-coated pits derived from clathrin-coated vesicles are not formed in hypertonic medium [38]. To this end, infected cells were incubated with anti-RSV IgG anti-RSV in hypertonic sucrose medium for different times (0, 10, 20, 30, 40, 50, or 60 min), and then to permeabilized fixed cells, anti-RSV IgG was added and analyzed by confocal microscopy.

**Figure 4:**
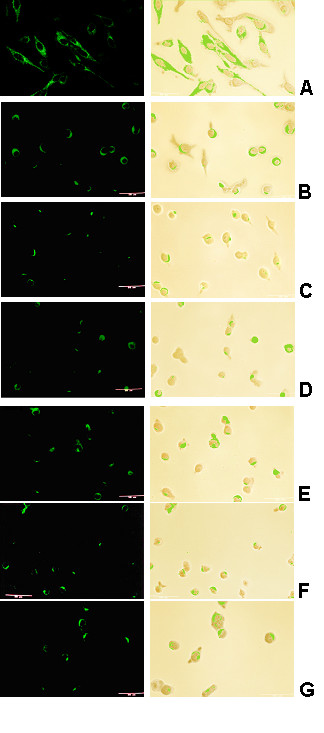
**Kinetics of intracellular RSV proteins in cells incubated in hypertonic medium**. HEp-2 cells had been infected with anti-RSV IgG and then were incubated for different times in sucrose medium permeabilized and fixed, in acetone and methanol. Confocal laser scanning was used to obtain images of intracellular viral proteins visualized as described in Figure 3.

As shown in Figure 4A (0 min), the characteristic epithelial cell morphology was evident and the cytoplasm was covered with fluorescently labelled viral proteins (intensity of 3), implying that viral proteins were homogenously distributed in the cytoplasm. Cell morphology changed after incubation in hypertonic medium: The cells became round and the concentration of viral proteins and their intra-cellular localization varied with incubation time. Furthermore, between 0 minutes and subsequent incubation times, a marked drop in the concentration of labelled proteins and in the fluorescence intensity were observed (Fig. 4A to 4G).

At 10-minute incubation, intracellular fluorescently labelled proteins were localized in one area of the cytoplasm of the rounded cells; patches with fluorescence (intensity of 1) were distributed randomly (Fig. 4B). At 20-minute incubation, a decrease in the concentration of RSV intracellular proteins was evident and the fluorescently labelled proteins (intensity of <1) were localized in patches and cells without detectable fluorescently labelled proteins were present (Fig. 4C). At 30-minute incubation, the content of fluorescently labelled viral proteins (intensity of 1) increased (Fig 4D). At 40-minute incubation, a noticeable decrease in fluorescent proteins was observed (Fig 4E); however, slightly higher fluorescence intensity was observed in the samples from the 50 (Fig. 4F) and 60-minute (Fig. 4G) incubations.

Viral proteins were continually present throughout the assays, suggesting that either clathrin-mediated endocytosis was inhibited or viral protein synthesis *de novo *took place. During the assays, the concentration of viral proteins varied, although the fluctuation was less pronounced than that in the former determinations (Fig. 2 and 3).

### Anti-RSV IgG protects HEp-2 infected cells from viral induced death

To evaluate whether the presence of anti-RSV IgG has an effect on cell viability, RSV- and mock-infected HEp-2 cells were incubated with either anti-RSV IgG or with IgG from pre-immune serum and the cell viability was determined at 2, 24, 48 and 72 h p.i. As control, mock-infected cells were incubated with anti-RSV IgG and pre-immune IgG.

Cell viability differed in infected cells incubated with anti-RSV IgG or with IgG from pre-immune serum. Viable-cell density remained constant when RSV-specific immunoglobulins were used; in contrast, in infected cells incubated with non-immune IgG, the viability of the HEp-2 cells was reduced and syncytia was observed (results not shown). In comparison to the value obtained at 2 h p.i., the cell survival at 48 and 72 h p.i. was reduced to 6.5% and 1.45%, respectively. No cell viability change was evident in mock-infected HEp-2 cells incubated with either anti-RSV IgG or pre-immune IgG. The data suggest that anti-RSV polyclonal IgG protected infected cells from viral-induced death.

## Discussion

In this report, we determined the effect of incubation RSV infected epithelial cells with polyclonal RSV IgG on expression and localization of viral proteins and cell viability. Kinetics of cell-surface viral glycoproteins and intracellular viral proteins was monitored through confocal microscopy and cell viability determined by trypan blue exclusion.

Our data show that the binding of specific antibodies to RSV glycoproteins anchored to the plasma membrane of human epithelial cells led to their redistribution and aggregation (Fig 2). Subsequently, complexes of aggregated viral glycoproteins with bound antibody became clustered in patches (Fig. 2B) that then formed caps (Fig. 2C) with concomitant fluctuations on RSV Ag-Abs concentration. (Fig. 2).

The lower concentration of RSV Ag-Abs on cell surface can be explained through cap formation, with subsequent release to the extracellular medium or by endocytosis of RSV Ag-Abs. Reduction of RSV Ag-Abs concentration on the cell-membrane was evidenced at both 30 and 50 minutes of incubation with anti-RSV IgG (Fig. 2C and 2E) implying that RSV Ag-Abs loss was done, either by caps being expelled to the extracellular space or patches being internalized by endocytosis (Fig. 4).

The interpretation that caps were released into the medium was supported by the simultaneous presence of cells with caps and areas without detectable viral glycoproteins (Fig. 2C). This observation agrees with reports for pseudorabies virus in which 17% of the caps were found to have been spontaneously expelled into the extra-cellular space, thus leaving behind cells with the above-mentioned characteristics [7]. However, with the methodology we used, it was not possible to conclude that caps were extruded. Therefore, studies are in progress to obtain definitive data.

Our data, obtained by incubation in hypertonic media, showed that the concentration of intracellular viral proteins decreased over the course of the determinations, thereby suggesting that endocytosis through clathrin-mediated mechanism was inhibited (Fig. 4). The remaining fluorescein-labelled viral proteins present in the cytoplasm during these assays (Fig. 4B to 4D) might have been due to protein synthesis *de novo *and/or RSV Ag-Abs internalized through a mechanism(s) different from clathrin-mediated endocytosis.

Protein synthesis *de novo *is particularly considered because, throughout the course of these experiments, we observed that infected cells incubated with anti-RSV IgG were protected from viral-induced death. This observation was confirmed through comparative cell-survival determinations between infected HEp-2 cells incubated either with anti-RSV IgG or with pre-immune IgG. The loss of cell- surface viral glycoproteins during culturing RSV infected cells for longer than 72 h in the presence of anti-RSV IgG might explain the lack of syncytia formation and hence avoid death of the cell [11].

The increase in RSV Ag-Ab concentration may be explained as resulting from the internalization of the RSV Ag-Abs, with subsequent dissociation of the complexes and recycling of the liberated viral antigen back to the cell surface [37] and/or of synthesis *de novo *of viral proteins

Our data suggest that in the human epithelial cell line we used both caps formation and endocytosis took place (Fig. 2 and 3). In contrast in pseudorabies virus capping or internalization initiated by cell-surface protein-specific antibody interaction depends on the cell type [5,6],

Although viral proteins on the cell plasma membrane were detected at m.o.i. of 10 to 20, the glycoprotein-antibody complexes were clearly observable as patches at higher multiplicity, implying that a defined content of RSV proteins was required for clustering into patches. This observation is similar to that of reports on monocytes infected with pseudorabies virus, in which glycoprotein capping occurred only after the patch size exceeded a minimal threshold size [6].

During consecutive assays, cell-surface- and intracellular-protein concentrations fluctuated in a cyclic manner, thus suggesting a continued removal and replacement of cell-surface and intracellular proteins. In the current work, determination was made at 10-minute intervals; however, by using either shorter or longer time intervals, the fluctuation cycles in the concentration of viral-protein-antibody complexes may be optimized.

Exactly how RSV Ag-Abs initiate the redistribution process, in capping or in internalization, is not fully understood; however, in the *alphaherpesvirinae *family has been reported that specific tyrosine family of motifs (YXXPHI; Y standing for tyrosine, X for any aminoacid and PHI for a hydrophobic residue), in the cytoplasmic tails of the viral transmembrane glycoproteins activate clathrin-mediated endocytosis [39]. Therefore, it is interesting to note that tyrosine YXXPHI motifs are present in cytoplasm residues of both the F and G glycoproteins of RSV [11].

The present findings indicate that specific antibody bounded to the surface of RSV infected cell modify cell-surface viral determinants, the intracellular viral polypeptide concentration and interfere with viral induced destruction of the cell.

Alterations of RSV intracellular viral proteins expression by anti-RSV IgG interaction with cell-surface viral glycoproteins is reminiscent with reports in the measles virus system, where expression of intracellular viral proteins is modify by the interaction of specific antibodies with cell-surface viral glycoproteins [5]. Measles virus like RSV is a member of the *Paramyxoviridae *family [40].

How these processes are involved in the viral life cycle and viral pathogenesis is unknown, however, increase in viral replication with concomitant enhance of the disease [17-19] might be related to RSV Ag-Abs induced delay on cell destruction. Furthermore, retrieval of RSV envelope proteins from the cell-plasma membrane lowers the amount of viral determinants that are exposed at the cell surface, and may therefore reduce the efficiency of recognition by the immune system favouring viral persistence in the organism [5,41]. Virus to persist must evade immune surveillance and not kill the host cell [42].

To our knowledge, this is the first report on antibody-induced modulation of respiratory syncytial virus antigens. Moreover, the system we described allows study long time interaction between RSV infected cells and antiviral antibodies.

## Abbreviations

RSV Respiratory Syncytial Virus

COPD Chronic Obstructive Pulmonary Disease

RSV Ag-Abs RSV-cell-surface-antigen-antibody complexes

## Competing interests

The author(s) declare that they have no competing interests.

## Authors' contributions

RES and RT contributed in the experimental work, analysis of results and discussion of results. LV performed the majority of the experimental work and BG conceived and wrote the manuscript. All authors read and approved the final manuscript
